# Transmastoid exposure of the labyrinthine segment of the facial nerve: an anatomical study^☆^^☆☆^

**DOI:** 10.1016/j.bjorl.2021.07.002

**Published:** 2021-08-11

**Authors:** Gulay Guclu Aslan, Asim Aslan, Selcuk Surucu

**Affiliations:** a Medical Sciences University, Tepecik Research and Training Hospital, ENT Clinic, Izmir, Turkey; b Manisa Celal Bayar University, Faculty of Medicine, Department of ORL, Manisa, Turkey; c Koc University, Faculty of Medicine, Department of Anatomy, Istanbul, Turkey

**Keywords:** Ear surgery, Anatomy, Facial nerve, Labyrinthine segment

## Abstract

**Introduction:**

Compression of the labyrinthine segment of the facial nerve by edema has been considered as an important pathology in the majority of the cases of idiopathic facial nerve paralysis. Hence, it is suggested that total decompression of the facial nerve should also include the labyrinthine segment by a middle fossa approach. However, the middle fossa approach requires craniotomy and temporal lobe retraction, which increases the morbidity. The labyrinthine segment of the facial nerve can also be reached through mastoidectomy. However, many ear surgeons are not familiar with this approach due to the lack of anatomical data on this surgical area.

**Objective:**

To study the anatomical limitations of decompression of the labyrinthine segment via transmastoid approach.

**Methods:**

Complete mastoidectomy was performed in six adult cadavers heads. Dissection was extended in the zygomatic root and posterior bony wall of the external auditory canal to visualize the incudomallear joint completely. The bone between tympanic segment, lateral and superior semicircular canal’s ampullas and middle fossa dural plate was removed. Fine dissection was carried out over tympanic segment of the facial nerve in an anterosuperomedial direction the labyrinthine segment was reached.

**Results:**

All the mastoids were well pneumatized. Distances between the labyrinthine segment and middle fossa dura, and between the labyrinthine segment and superior semicircular canal, were 2.5 and 4.5 mm on average, respectively. In addition, distances between the middle fossa dura and dome of the lateral semicircular canal, and between the middle fossa dura and tympanic segment were 4.6 mm and 4.3 mm on average, respectively.

**Conclusion:**

It is possible to expose the labyrinthine segment of the facial nerve through mastoidectomy by dissecting the bone in the area between the tympanic segment of the facial nerve, middle fossa dural plate and ampullary ends of the lateral and superior semicircular canals.

## Highlights

• Surgical decompression of the labyrinthine segment of the facial nerve is important.
• The main approach is the middle fossa approach, which has serious morbidities.
• It is also possible to reach the labyrinthine segment by transmastoid approach.
• Detailed anatomic knowledge about surgical area for the transmastoid approach is essential.

## Introduction

The labyrinthine segment (LS) of the facial nerve (FN) is the shortest and narrowest part of the FN within the temporal bone.^1^, ^2^, ^3^ Also within the labyrinthine segment of the facial canal, the ratio of spatial occupancy of the main vessels to the canal and to the nerve was found to be smaller comparing in the tympanic and mastoid segment.^4^ All these anatomical features make LS vulnerable to compression by edema due to inflammation in idiopathic facial nerve paralysis, i.e., Bell’s palsy.^5^, ^6^, ^7^, ^8^, ^9^ When medical treatment is not successful or poor prognostic electrophysiological findings exist, surgical decompression of the facial nerve including the LS is suggested.^6^, ^7^, ^8^, ^9^, ^10^ In this type of surgery, the mastoid and tympanic segments of the FN are decompressed through mastoidectomy while the LS is decompressed via a middle fossa approach.^5^, ^7^, ^8^, ^11^^,^^12^ However, this surgery requires wide skin incision, craniotomy and temporal lobe retraction for surgical exposure, which may lead to complications such as seizures, cerebrospinal fluid leak and sensorineural hearing loss.^5^, ^7^, ^8^, ^12^^,^^13^ If the LS of the FN can be reached through mastoidectomy, surgical morbidities of the middle fossa approach can be avoided.^14^, ^15^, ^16^, ^17^, ^18^, ^19^ Available anatomic studies mainly concentrate on how much the LS can be exposed after transmastoid dissection by measuring the length of the LS.^20^, ^21^ However, it is more important for inexperienced surgeons to know the dimensions of the surgical area between anatomical structures around the LS.

This anatomical study was planned to demonstrate anatomical limitations of transmastoid approach to reach the LS of the FN.

## Methods

The study was approved by the Biomedical Ethics Committee of Koc University (2018.202.IRB2.035). The study was conducted on six adult cadavers’ heads, of which 4 were women and 2 were men. The mean chronological age of the specimens was 79 (65–92).

### Dissection method

**Figure 1:**
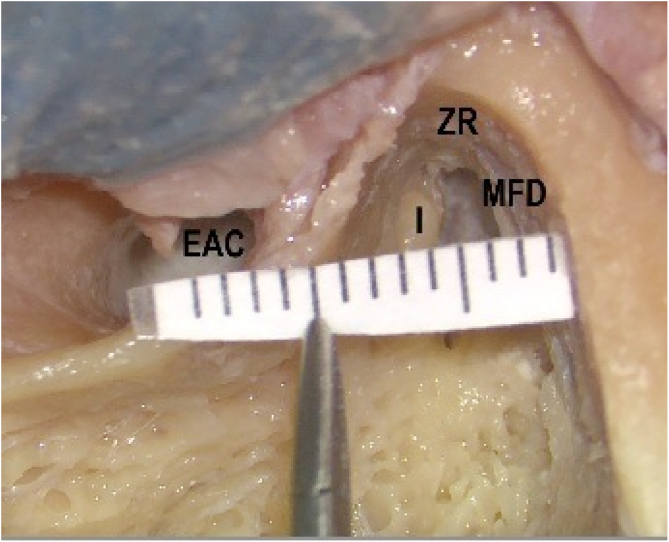
Complete mastoidectomy on the left temporal bone. Extended dissection in the zygomatic root (ZR) and posterior bony wall of the external auditory canal (EAC). Middle fossa dural plate (MFD) was exposed by drilling the bone over it (I, Incus).

**Figure 2:**
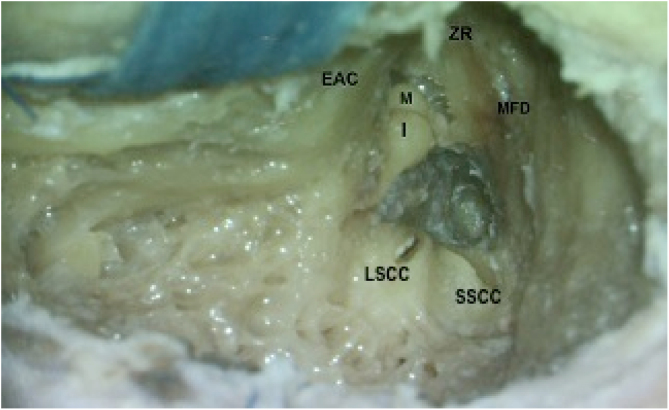
Posterior bony wall of the external auditory canal (EAC) was thinned. Body of the Incus (I) and head of the malleus (M) are visualized. Bone between the tympanic segment of the facial nerve (TS), ampullary ends of lateral semicircular canal (LSCC) and superior semicircular canal (SSCC), and MFD were removed (ZR, Zygomatic root).

**Figure 3:**
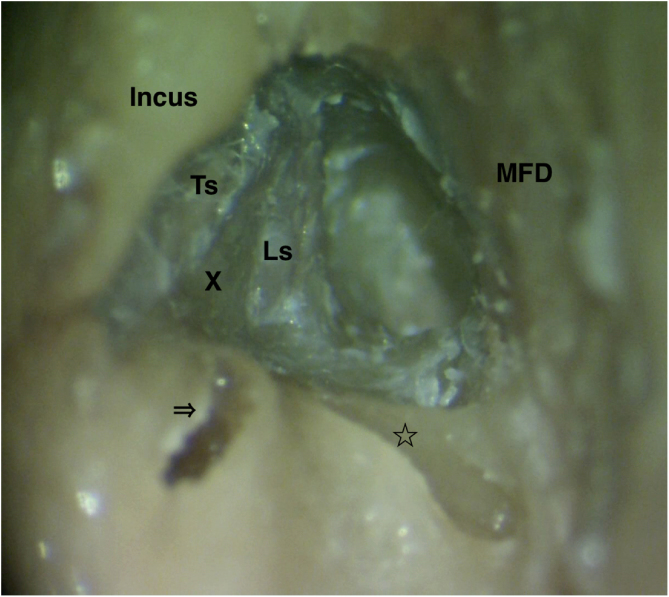
Closer view of the labyrinthine segment (Ls) after bone removal between tympanic segment (Ts), ampullary ends of the lateral semicircular canal (arrow) and superior semicircular canal (star), and MFD. For better demonstration membranous labyrinths of both semicircular canals were removed. X, Vertical crest at the fundus of the internal acoustic canal.

Following complete mastoidectomy, dissection was extended in the zygomatic root, and posterior bony wall of the external auditory canal (EAC) was thinned to visualize the incudomallear joint (IMJ) completely (Fig. 1). Pneumatization of the mastoid bone was judged as well pneumatized if all air cells were filled with air, and as poorly pneumatized if air cells contained soft tissue or if no visible air cells existed, i.e., sclerotic. The middle fossa dura (MFD) was exposed by drilling the bone over it. The bone between TS, ampullary ends of lateral semicircular canal (LSCC) and superior semicircular canal (SSCC), and MFD were removed (Figure 2, Figure 3). Fine dissection was carried out over TS of the FN in an anterosuperior direction towards the cohleariform process. Dissection was continued in a medial direction to expose the LS.

### Measurements

**Figure 4:**
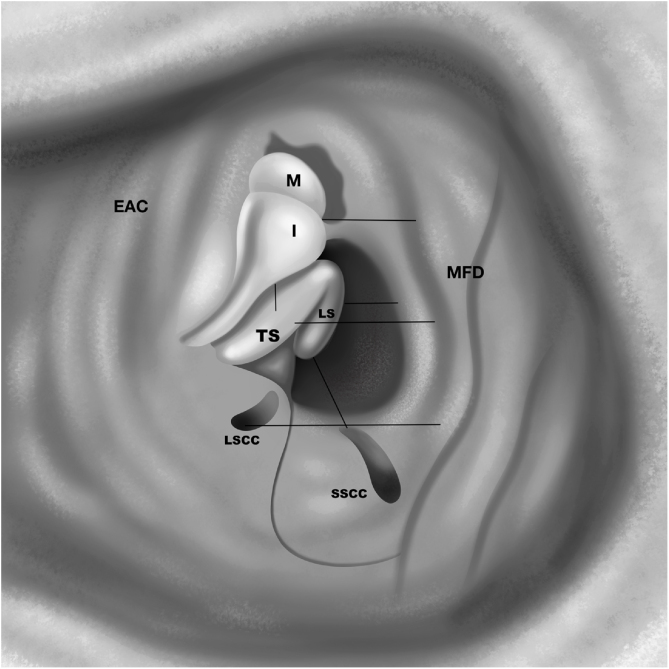
Drawing of the anatomic measurements. EAC, external auditory canal; MFD, Middle fossa dural plate; I, Incus; M, Malleus; TS, Tympanic segment of the facial nerve; LS, Labyrinthine segment of the facial nerve; LSSC, Lateral semicircular canal; SSSC, Superior semicircular canal.

All measurements were done by millimetric scale paper (Fig. 1). Following measurements were done (Figure 1, Figure 4):

1) MFD to EAC (at the point of Henle’s Spine),
2) MFD to dome of the LSSC,
3) MFD to IMJ,
4) MFD to TS,
5) MFD to LS
6) LS to SSCC ampulla,
7) IMJ to TS.

## Results

All the mastoids were well pneumatized. Measurements related to dissection to expose the LS of the FN were demonstrated in Table 1. Distances between LS and MFD, and between LS and SSC, were 2.5 and 4.5 mm on average, respectively. In addition, distances between MFD and dome of LSCC, TS were 4.6 mm and 4.3 mm on average, respectively.

**Table 1:** Measurements of the dissection.

	Cad. 1		Cad. 2		Cad. 3		Cad. 4		Cad. 5		Cad. 6		Average (mm)
	R	L	R	L	R	L	R	L	R	L	R	L	
**MFD - EAC**	10	11	10	9	8	8	10	13	9	8	11	13	10
**MFD - LSCC**	5	4	5	7	4	5	5	6	3	3	6	3	4.67
**MFD - IMJ**	6	5	5	6	5	5	5	8	3	4	6	4	5.17
**MFD - TS**	5	4	4	4	5	3	4	5	3	4	5	4	4.33
**MFD - LS**	3	2	1	2	3	2	3	3	2	3	4	2	2.5
**LS - SSCC**	4	5	5	5	4	3	3	4	4	5	3	5	4.58
**IMJ - TS**	2	2	2	2	2	2	3	4	3	3	3	3	2.58
	**Female**		**Male**		**Female**		**Female**		**Female**		**Male**		
**Age of death**	83		82		71		92		81		65		79

MFD, Middle Fossa Dura; EAC, External Auditory Canal; LSCC, Lateral Semicircular Canal; IMJ, Incudomallear Joint; TS, Tympanic Segment of the facial nerve; LS, Labyrinthine Segment of the facial nerve; SSCC, Superior Semicircular Canal.

## Discussion

Surgical treatment in cases of FN paralysis caused by inflammation, such as Bell’s palsy, herpes zoster oticus etc., is recommended either when the medical treatment fails or poor prognostic factors exist.^5^, ^6^, ^7^, ^8^, ^9^ The basic approach is decompression of the mastoid and tympanic segments of the FN within the temporal bone.^22^ However, electrophysiological and histologic findings and earlier surgical results indicate that the main pathology in those cases present primarily in the LS of the FN.^5^, ^6^, ^7^, ^8^, ^9^, ^10^, ^11^, ^12^ These data led to the idea that decompression should also include the LS. Presently, the most accepted popular approach to decompress the LS is via a middle fossa approach.^5^, ^6^, ^7^, ^8^, ^9^, ^11^, ^12^ Nevertheless, this type of surgery has potentially life-threatening morbidities, including seizures, sensorineural hearing loss and cerebrospinal fluid leak although the outcome for such cases i.e., Bell’s palsy and herpes zoster oticus, are not life threatening.^13^, ^14^, ^15^ From this point of view, it seems that such a radical approach is not appropriate for non-critical disorders.

### Decompression by transmastoid approach

In 1979, May noted that Salaverry had reported that the LS of the FN could be approached through a trans mastoid approach.^14^ However, this approach was not popular, and failed to attract the attention of surgeons.^15^, ^16^, ^17^, ^18^, ^19^ This is mainly due to the fact that anatomically it is difficult to reach the area and inadequate anatomical data exists about surgical exposure of the LS throu gh atrans- mastoid approach. Such data facilitates learning this type of surgery, especially for inexperienced surgeons.

### Mastoid pneumatization

Since the LS is located deep to the epitympanum, and fine surgical manipulation is required to expose the LS, obtaining wider surgical exposure on the lateral mastoid surface is an essential step for safe surgery. Temporal bone pneumatization has a critical role in this respect. It has been reported that the amount of exposure of the LS depends on the temporal bone pneumatization extent.^23^ All specimens in our study had good mastoid pneumatization. In all specimens, the distance between the EAC and MFD was 10 mm, which is quite enough to dissect the surgical area at the medial wall of epitympanum. However, it should be kept in mind that since the dimension of the surgical area is possibly smaller in sclerotic or semi-sclerotic mastoids, the dissection around the LS might be difficult. New studies are required to examine the anatomical dimensions of this type of surgery in non-pneumatized mastoids.

### Chronological age

It can be considered that transmastoid access to the LS of the FN might be difficult, since the temporal bones have smaller dimensions in children under 13–17 y.o than in adults. In the present study, the average chronological age of the specimens was 79 y.o, and the anatomical dimension of surgical area was found sufficient for dissection. However, since there were no specimens of childhood age group in our study, we cannot comment on the dimensions of the surgical area in this age group.

### Surgical manipulation around ossicles

In addition, we observed that bone removal on the EAC until adequate exposure of the IMJ, and on the zygomatic root until exposing the Cogs area is quite useful and makes it easier to dissect the bony area between the TS and MFD. Such surgical exposure leads to safer dissection to avoid tsurgical trauma to the IMJ.

Previous reports suggest dislocation of the ossicles to decompress the TS, which may lead to conductive hearing loss postoperatively.^14^, ^15^ We observed that decompression of the TS could be managed without dislocating ossicles. Decompression of the TS could be done by drilling on the superior part of the tiny bone over the TS. Fine hooks can be used for removal of the rest of the bone on this segment. The distance between IMJ and TS is 2 mm, which permits safe surgical manipulation over that segment.

### Finding the labyrinthine segment

After dissection over the TS is followed to reach the geniculate ganglion, the drilling is made on the medial direction to find LS. In order to increase the surgical exposure, it is important to remove the bone superiorly to the TS up to the MFD, and between LS and ampullary ends of the SSCC and LSCC.^17^ We found that this area was very narrow and required very fine dissection (Figure 2, Figure 3) (Table 1). For safe dissection in this area, it is necessary to know anatomical relationship of LS with ampullary ends of both semicircular canals. The distance between the LS and ampullary end of the SSCC was found to be 4.5 mm on average, which is consistent with findings of previous studies.^13^ Changing the position of the head towards to the surgeon, it is possible to dissect the total length of the LS in pneumatized bones (Fig. 3). This is important because it is reported that the narrowest part of the LS is at its meatal foramen end,^2^, ^3^ and it is claimed that the edema of that area is mainly responsible from the nerve conduction block in cases of idiopathic FN paralysis.^6^, ^7^, ^8^, ^9^, ^10^ Hence it is important to expose the nerve in that area to manage decompression of the nerve.

### Dura exposure

It was found that the distance between MFD and LS was 2.5 mm on average. To expose the LS sufficiently, the bony dural plate should be drilled, which means exposure of the middle fossa dura. In such situation dural exposure should be minimized as much as possible because wider exposure of dura runs the risk of dural laceration and complications, as chronic otorrhea or meningocele in the long term. Hence dissection on this area requires much attention and care.

### Limitations of the study

It is well known that the temporal bone has many anatomical variations such as pneumatization, position of the MFD, sigmoid sinus position etc., which may restrict the surgical exposure on it.^24^, ^25^ This is especially important for transmastoid exposure of the labyrinthine segment of the FN. Poor pneumatization, lower located MFD and an anteriorly placed sigmoid sinus might make it difficult or even sometimes impossible to expose the LS via mastoidectomy. Because of the small sample size (6 cadavers, 12 sides) in the present study, the effect of such variations on the surgical exposure could not be studied. Hence, the conclusions of the present study should not be extended to all situations.

## Conclusion

Decompression of the LS is possible through the transmastoid approach by dissecting the bone on the area between tympanic segment of the FN, middle fossa dural plate and ampullary ends of the lateral and superior semicircular canals. However, it should be done by the experienced surgeon who has good thorough knowledge about the anatomical relationships in the surgical field.

## Conflicts of interest

The authors declare no conflicts of interest.
